# A reflection on COVID-19 and oral mucosal lesion: a systematic review

**DOI:** 10.3389/froh.2023.1322458

**Published:** 2023-12-18

**Authors:** Wai Ying Kot, Jing Wen Li, Alice Kit Ying Chan, Li Wu Zheng

**Affiliations:** ^1^ Faculty of Dentistry, The University of Hong Kong, Hong Kong, Hong Kong SAR, China; ^2^ Division of Oral & Maxillofacial Surgery, Faculty of Dentistry, The University of Hong Kong, Hong Kong, Hong Kong SAR, China; ^3^ Division of Restorative Dental Sciences, Faculty of Dentistry, The University of Hong Kong, Hong Kong, Hong Kong SAR, China

**Keywords:** oral mucosal lesions, oral lesions, COVID-19, SARS-CoV-2, oral manifestations, systemic review

## Abstract

**Introduction:**

The aim of this systematic review is to provide a clinical update of the current knowledge on COVID-19 and oral mucosal lesions, to analyze the types and prevalence of oral mucosal lesions in patients with COVID-19, and to clarify the potential association between COVID-19 and oral mucosal lesions.

**Methods:**

The literature search was conducted using PubMed, Web of Science, Scopus and the Cochrane Library, as well as literatures via manual searches of the reference lists of included studies. Studies published in English that mentioned oral mucosal lesions in patients with COVID-19 were included, resulting in a total of 31 studies.

**Results:**

Most of the included studies were considered to have a moderate to high risk of bias according to the Joanna Briggs Institute bias assessment tools. Based on COVID-19 severity, the characteristics and patterns of oral mucosal lesions in COVID-19 patients were described, analyzed and synthesized. Overall, ulcers without specific diagnosis had the highest prevalence in COVID-19 patients, followed by traumatic ulcers, candidiasis, petechiae and aphthous-like lesions. Homogeneity of data cannot be achieved in statical analysis, indicating randomness of outcome (ulcers without specific diagnosis, 95% CI: 28%–96%, *I*^2^ = 98.7%).

**Discussion:**

Given the limited evidence from currently available studies, the association between COVID-19 and oral mucosal lesions remains difficult to clarify. Healthcare professionals should be aware of the possible association between COVID-19 and oral mucosal lesions, and we hereby discuss our findings.

## 1. Introduction

COVID-19, a pandemic that emerged in December 2020, has since affected more than 600 million people and caused more than 6 million deaths. Fever, cough, fatigue and loss of smell or taste have been reported as the most common symptoms of COVID-19. In severe cases, critical illness with pneumonia or even respiratory failure has been demonstrated (1).

Current research suggests that Angiotensin-converting enzyme 2 (ACE2), a functional receptor on cell surfaces, is the entry point of SARS-CoV-2 into the host cell (2). It is highly expressed in oral mucosa, especially in tongue epithelial cells, allowing virus invasion via the oral cavity. Thus, these previous findings indicate that oral mucosa may be a target for the SARS-CoV-2.

Therefore, COVID-19 may have potential manifestations in the oral cavity. Oral signs and symptoms of COVID-19 have been reported (3–24). Among these signs and symptoms, lesions of the oral mucosa have been commonly demonstrated. These include ulceration, desquamative gingivitis, petechiae and co-infections such as candidiasis and recurrent HSV. The mechanisms underlying the relationship between oral mucosal lesions and COVID-19 are not yet clear and well understood. It is still controversial whether they are a consequence of direct viral infection, systemic deterioration, adverse effects of medical treatments, or coincidence (5, 6, 10–12, 15, 24–27).

The mechanisms underlying the relationship between oral mucosal lesions and COVID-19 are not yet clear and well understood. It is still controversial whether they are a consequence of direct viral infection, systemic deterioration, adverse effects of medical treatments, or coincidence (5, 6, 10–12, 15, 24–27). The effect of COVID-19 on the oral cavity has been noted in previous review articles (3, 16, 18–21, 23, 24, 28–30). However, only a few of them shed light on oral mucosal lesions. As such, clinical update is needed to obtain a better understanding of oral mucosal lesions presented in COVID-19 cases.

In this context, a systematic review has been conducted. The objective of this review is to perform a clinical update of the current knowledge about COVID-19 and oral mucosal lesions, analyze the types and prevalence of oral mucosal lesions in patients with COVID-19, and clarify the potential association between COVID-19 and oral mucosal lesions.

## 2. Method

### 2.1. Study protocol

PRISMA-P (PRISMA for protocols) guideline (31) was followed for a systematic and comprehensive approach. The systemic review was registered at the International Prospective Register of Systematic Reviews (PROSPERO) database under registration code CRD42022377790.

### 2.2. Eligibility criteria

Inclusion and exclusion criteria were defined as follows, consisting of observational studies investigating the prevalence of oral mucosal lesions in patients with COVID-19.

The inclusion criteria were: (1) case reports, case series, cross-sectional studies and cohort retrospective studies; (2) report oral mucosal findings in PCR-confirmed cases of COVID-19; and (3) publish between January 2020 and August 2022.

Exclusion criteria were: (1) non-English studies; (2) lack of description of oral mucosal findings; and (3) cannot access to the full article.

### 2.3. Information source and search strategy

Electronic searches were performed in PubMed, Web of Science, Scopus and Cochrane Library databases. The following combination of MeSH and non-MeSH keywords were used: “COVID-19”, “SARS-CoV-2”, “2019 NCOV”, “oral mucosal lesions”, “oral lesions”, “oral ulcer”.

An additional search of the literature was performed by manual search across reference lists of included studies. EndNote 20 was used to collect references and remove duplicate articles. Covidence.org was used to select studies and retrieve data.

### 2.4. Study selection

The selection was conducted in two phases. In phase 1, two authors (W.Y.K and L.W.Z) independently reviewed the titles and abstracts of all relevant references. In phase 2, the same selection criteria were applied to the full-text articles independently by the same 2 authors, ensuring that all the eligibility criteria were met. The final selection was always based on the full text of the publication.

### 2.5. Data collection

Initially, the necessary information from the selected articles was collected by two authors (W.Y.K and L.W.Z). Any differences of opinion were resolved through discussion and mutual agreement between the two authors.

### 2.6. Risk of bias assessment

The Joanna Briggs Institute's Critical Appraisal Checklist (32–35) was used by two authors to check the risk-of-bias of all included case reports, case series, cross-sectional studies and cohort retrospective studies. A study was classified as having a high risk of bias when it reached a “yes” score of up to 49%, moderate when 50%–69%, and low when >70%.

### 2.7. Summary measures

As the severity of COVID-19 progresses, the immune system of the infected patient may be altered, which may contribute to the different presentation patterns of oral mucosal lesions in COVID-19 patients. To explore the possible association between COVID-19 severity and oral mucosal lesions, the results were divided into 3 groups based on COVID-19 severity: self-monitoring patients as mild cases, hospitalized patients as moderate cases, and intensive care unit (ICU) patients as severe cases. For all three groups, secondary outcomes included the clinical presentation and prevalence of oral mucosal lesions.

### 2.8. Synthesis result

Qualitative and quantitative synthesis was performed by sorting and comparing data extracted from the included studies on primary and secondary outcomes. Analyses of the proportions of oral mucosal lesions at different COVID-19 severity levels were calculated using Microsoft Excel software. The prevalence of non-specific diagnosed ulcers in studies with more than one case was expressed through relative or absolute frequencies and 95% CI, and a statistical analysis was performed using Stata. The *I*^2^ test was used to calculate statistical heterogeneity, which defined whether a fixed (*I*^2^ < 50%) or random (*I*^2^ ≥ 50%) effect model should be applied.

## 3. Result

### 3.1. Study selection and characteristics

**Figure 1:**
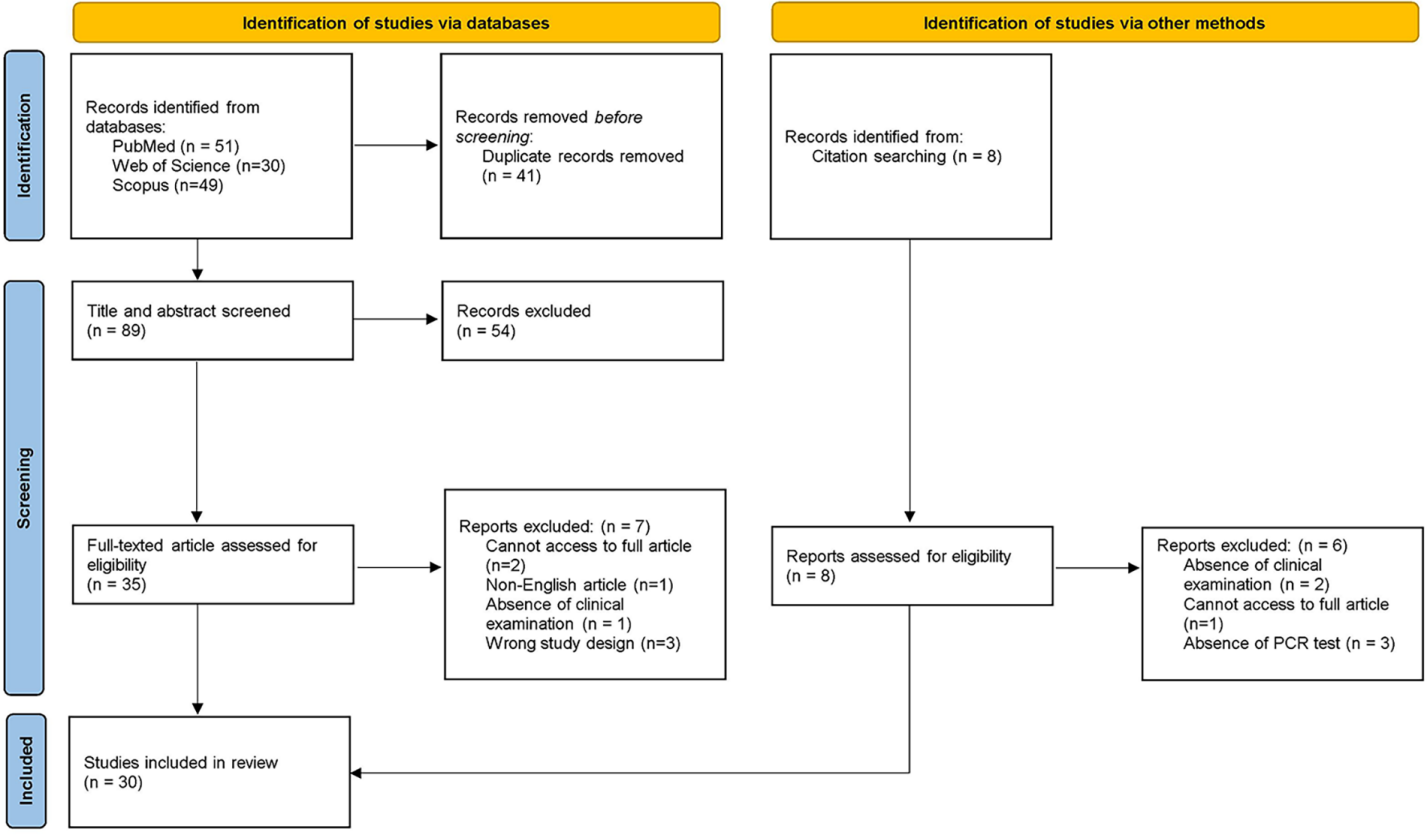
Flow diagram of literature search and selection criteria adapted from PRISMA.

In the first phase, 130 studies were identified from databases. After the removal of duplicates, 89 references remained for title and abstract screening. After the evaluation of all records, 35 articles remained for the second phase. A full-text reading was conducted, and 7 studies were excluded according to pre-defined eligibility criteria. The reference lists of all included articles were assessed and 8 were selected for full-text analysis. 6 articles were excluded according to the eligibility criteria. Thereafter, 30 studies were selected for result synthesis and statistical analysis. A flowchart describing the process is illustrated in Figure 1.

The included studies were distributed worldwide, with a higher prevalence in Europe. 11 studies were conducted in Europe, 7 in Asia, 2 in North America, 2 in Africa, and 8 in Latin America (Table 1). Within the included studies, 15 were case reports, 10 were case series, 4 were cross-sectional studies, and 1 was a retrospective study.

**Table 1:** Descriptive characteristics of included primary studies (*n* = 27).

Study ID	Study conducted country/area	Study design	Sample size	Population age (mean)	Population gender (M)	Population gender (F)	Covid severity	Risk of bias
Ansari et al. (36)	Iran	Case report	2	65.5	1	1	Moderate (hospitalised)	Moderate
Brandao et al. (37)	Brazil	Case series	8	53.875	5	3	Mild (self-monitoring); moderate (hospitalised); severe (ICU)	Low
CebeciKahraman and Caskurlu (38)	Turkey	Case report	1	51	1	0	Mild (self-monitoring)	Moderate
Ciccarese et al. (39)	Italy	Case report	1	19	0	1	Moderate (hospitalised)	Moderate
Corchuelo and Ulloa (40)	Unite state	Case report	1	40	1	0	Mild (self-monitoring)	Moderate
CruzTapia et al. (41)	Colombia	Case series	4	47.25	1	3	Mild (self-monitoring); moderate (hospitalised)	Moderate
Demirbas et al. (42)	Turkey	Case report	1	37	0	1	Moderate (hospitalised)	Low
DiazRodriguez et al. (43)	Spain	Case series	3	58	1	2	Mild (self-monitoring); moderate (hospitalised)	Moderate
Dominguez-Santas et al. (44)	Spain	Case series	4	33	3	1	Mild (self-monitoring); moderate (hospitalised)	Moderate
Eduardo et al. (45)	Brazil	Retrospective study	472	NR	322	150	Severe (ICU)	Moderate
EghbaliZarch and Hosseinzadeh (46)	Iran	Case report	1	56	0	1	Mild (self-monitoring)	Moderate
Favia et al. (47)	Italy	Cross sectional study	123	72	70	53	Moderate (hospitalised); severe (ICU)	Low
Fidan et al. (48)	Turkey	Cross sectional study	74	45.6	49	25	Mild (self-monitoring)	Moderate
Gabusi et al. (49)	Italy	Case report	1	78	1	0	Moderate (hospitalised)	Low
Garcez et al. (50)	Brazil	Case report	1	65	0	1	Severe (ICU)	Moderate
Glavina et al. (51)	Croatia	Case report	1	40	0	1	Mild (self-monitoring)	Moderate
Hockova et al. (52)	Slovakia	Case series	3	64.3	3	0	Severe (ICU)	Moderate
Indu (53)	India	Case report	1	NR	1	0	Moderate (hospitalised)	High
Jimenez-Cauhe et al. (54)	Spain	Case series	4	66.75	0	4	Moderate (hospitalised)	High
Jimenez-Cauhe et al. (55)	Spain	Case series	6	NR	2	4	Moderate (hospitalised)	Moderate
Katz and Yue (56)	United state	Cross sectional study	6	NR	0	6	Moderate (hospitalised)	Moderate
Kitakawa et al. (57)	Brazil	Case report	1	20	0	1	Mild (self-monitoring)	Moderate
MartinCarreras-Presas et al. (58)	Spain	Case series	1	65	0	1	Moderate (hospitalised)	High
Palaia et al. (59)	Italy	Case report	1	30	0	1	Mild (self-monitoring)	Low
Rai et al. (60)	India	Case report	2	67	1	1	Moderate (hospitalised)	Low
Riad et al. (61)	Egypt	Case series	13	51.08	5	8	Mild (self-monitoring); moderate (hospitalised)	High
Riad et al. (62)	Egypt	Case series	26	36.81	9	17	Mild (self-monitoring)	Moderate
Sinadinos et al. (63)	United Kingdom	Case report	1	65	0	1	Moderate (hospitalised)	High
Soares et al. (64)	Brazil	Case report	1	42	1	0	Moderate (hospitalised)	Moderate
Soares et al. (65)	Brazil	Case report	1	23	0	1	Mild (self-monitoring)	Moderate
Villarroel-Dorrego et al. (66)	Venezuela	Cross-sectional study	55	51	30	25	Moderate (hospitalised); severe (ICU)	Moderate

### 3.2. Risk of bias in studies

Risk-of-bias assessment in individual studies is summarized in Table 1. Case reports, case series, cross-sectional studies and retrospective cohort studies were evaluated using the Joanna Briggs Institute Critical Appraisal Checklist for each study design (32–35). Most of the included studies were case reports (16/31) or case series (10/30) with moderate (20/30) to high (5/30) risk of bias. Only 6 studies were at low risk of bias.

### 3.3. Result of syntheses

A total of 820 patients with oral mucosal lesions were included in this systemic review. A higher proportion of males was observed in the study, of which 507 were male, 313 were female. The mean age of the patients ranged from 19 to 78 years. All patients had obtained positive PCR tests for SARS-COV-2 virus. 14 studies reported mild COVID-19 cases, 19 studies were included for moderate COVID-19 cases, and 6 studies demonstrated severe COVID-19 cases. Demographic characteristics of included patients are provided in Table 1.

#### 3.3.1. Characteristics of oral mucosal lesions

Considering that the definition of COVID-19 severity was variable among included studies, we re-grouped patients according to categorization terms reported by authors. The characteristics of oral mucosal lesions were first classified according to the COVID-19 severity of the patient, and then further subclassified according to the diagnosis/clinical presentation of the lesion.

**Figure 2:**
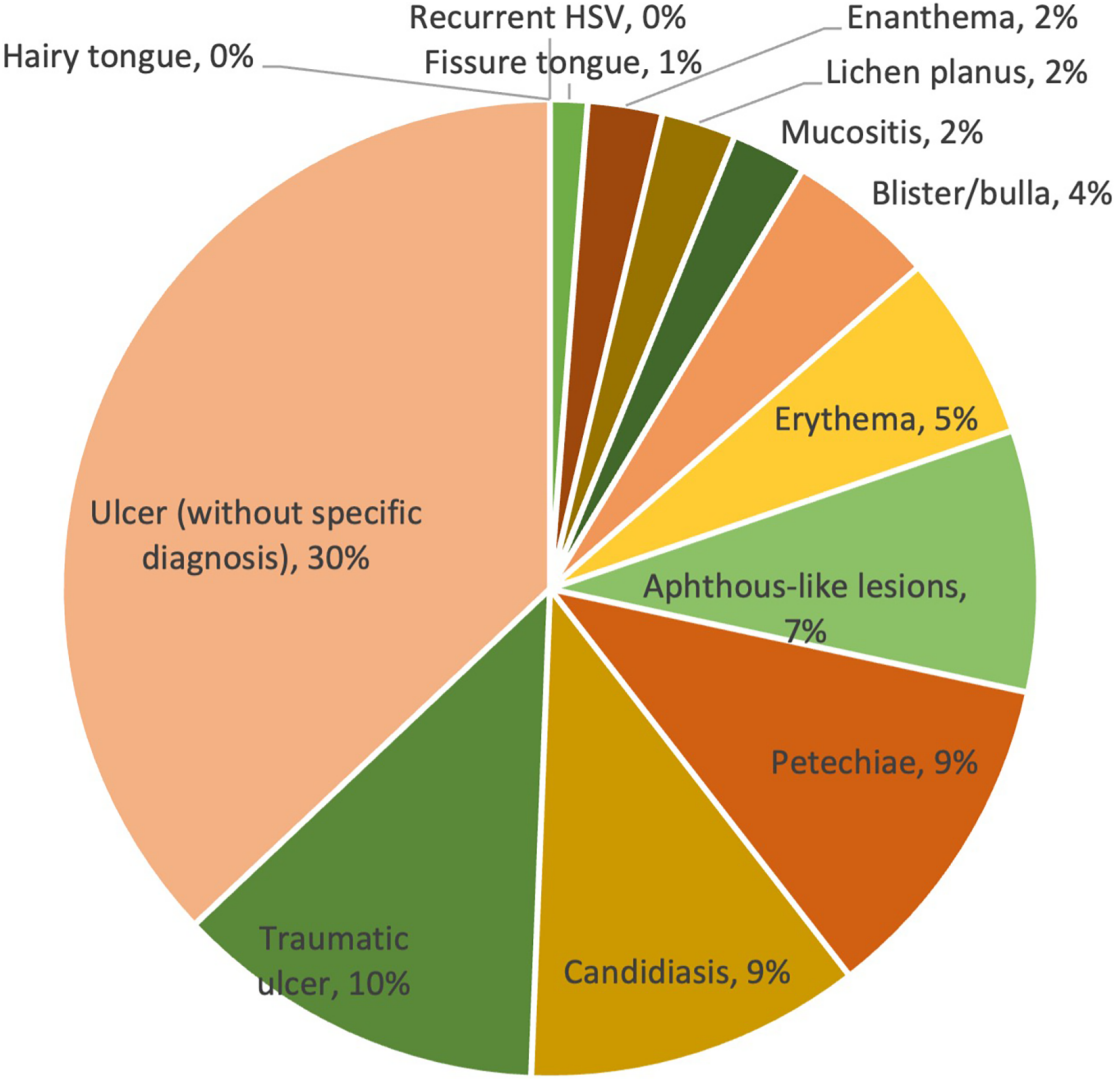
Overall prevalence of lesions in COVID-19 cases.

Overall, the prevalence of lesions ranged from 0% to 30%. The majority of lesions were ulcers without a specific diagnosis, reported in 30% of cases. Traumatic ulcers (10%) were the second most frequent lesions observed in patients with COVID-19, followed by petechiae (9%), candidiasis (9%) and aphthous-like lesions (7%). Other less commonly reported lesion diagnosis/clinical presentations were erythema (5%), blister/bulla (4%), enanthema (2%), lichen planus (2%), mucositis (2%) and fissure tongue (1%). The prevalence of lesion diagnosis/clinical presentation is shown in Table 2 and Figure 2.

**Table 2:** Prevalence of oral mucosal lesion diagnosis/clinical presentation in COVID-19 patients.

		Hairy tongue	Recurrent HSV	Fissure tongue	Enanthema	Lichen planus	Mucositis	Blister/bulla	Erythema	Aphthous-like lesions	Petechiae	Candidiasis	Traumatic ulcer	Ulcer (without specific diagnosis)	Participant number
Mild	Sum	1	1	0	1	12	9	3	19	32	2	0	0	36	126
	Prevalence	1%	1%	0%	1%	10%	7%	2%	15%	25%	2%	0%	0%	29%	100%
Moderate	Sum	0	0	4	8	0	4	17	11	8	6	26	0	85	189
	Prevalence	0%	0%	2%	4%	0%	2%	9%	6%	4%	3%	14%	0%	45%	100%
Severe	Sum	0	0	1	0	0	0	5	1	1	45	28	60	54	277
	Prevalence	0%	0%	0%	0%	0%	0%	2%	0%	0%	16%	10%	22%	19%	100%
Overall	Sum	1	1	5	9	12	13	25	31	41	53	54	60	175	592
	Prevalence	0%	0%	1%	2%	2%	2%	4%	5%	7%	9%	9%	10%	30%	100%

#### 3.3.2. Mild COVID-19 cases

**Figure 3:**
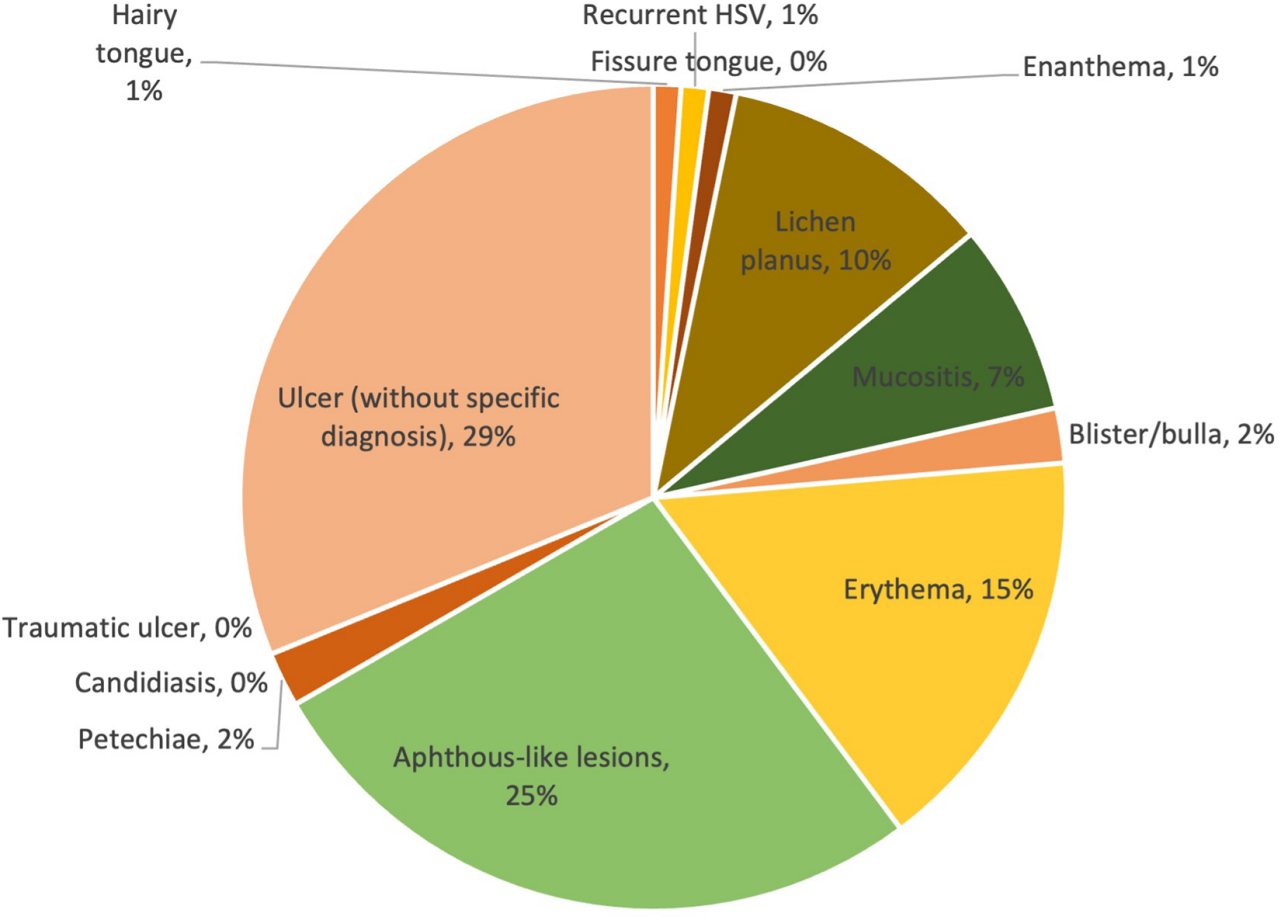
Prevalence of lesions in mild COVID-19 cases.

Of the 125 mild COVID-19 cases, the prevalence of lesion diagnosis/clinical presentation ranged from 0% to 29%. With a prevalence of 29% and 25% respectively, ulcers without specific diagnosis and aphthous-like lesions were two of the most prevalently reported oral mucosal lesions. The other types of lesions had a relatively low prevalence in comparison with the first two, with a prevalence equal to or less than 15%. The prevalence of lesion diagnosis/clinical presentation is shown in Figure 3.

#### 3.3.3. Moderate COVID-19 cases

**Figure 4:**
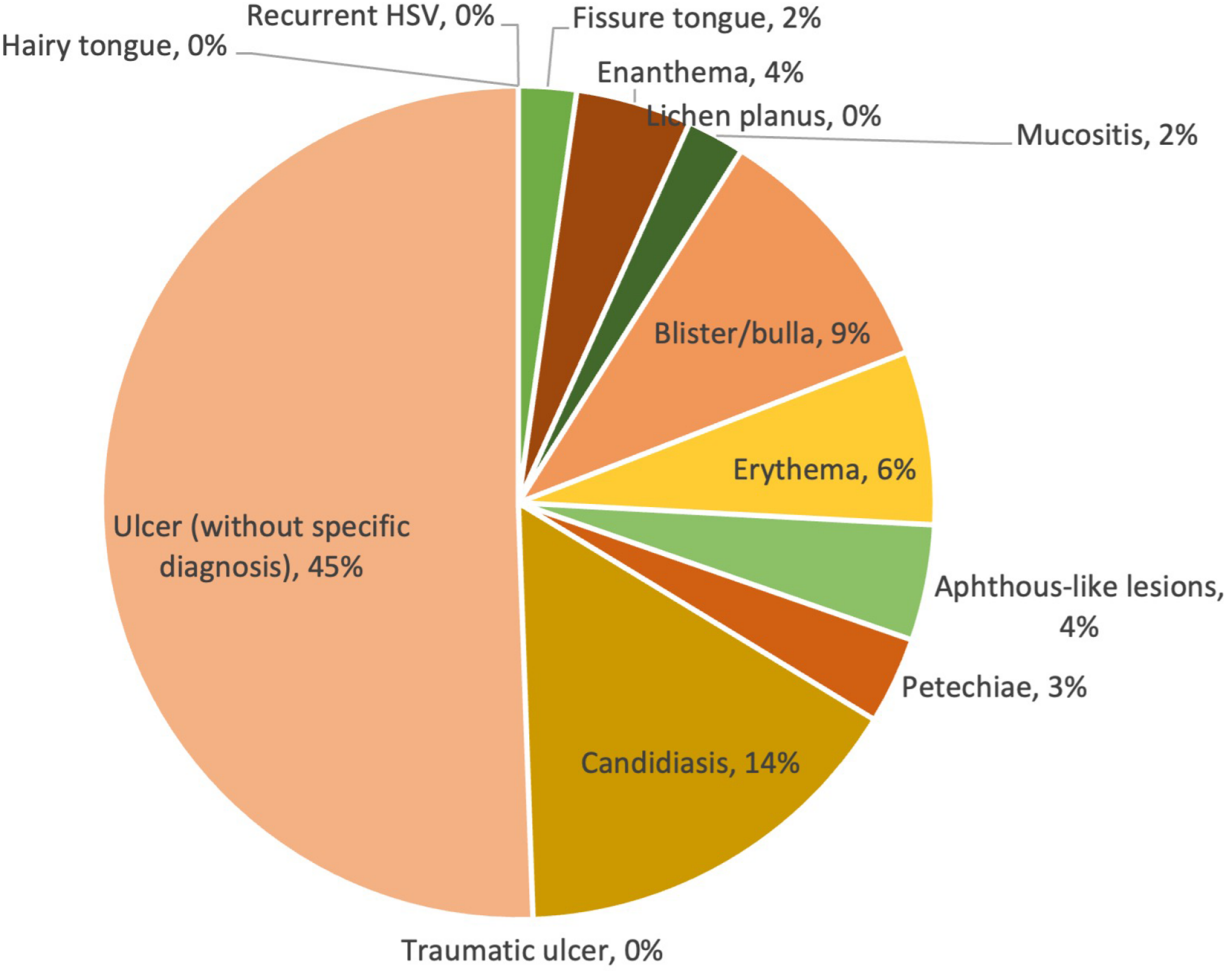
Prevalence of lesions in moderate COVID-19 cases.

Similar to the mild COVID-19 group, ulcers without specific diagnosis were still the most commonly reported lesion type. However, it showed a much higher prevalence in the moderate group (45%) than in the mild group (29%). It should be noted that although no cases of candidiasis were reported in the mild group, 26 cases (14%) were reported in the moderate group, being the second most common type of lesion in this group; whereas the aphthous-like lesions decreased dramatically from 25% (mild) to 4% (moderate). Other types of lesions had a relatively low prevalence compared to the first two, reaching a prevalence of less than 10%. The prevalence of lesion diagnosis/clinical presentation is shown in Figure 4.

#### 3.3.4. Severe COVID-19 cases

**Figure 5:**
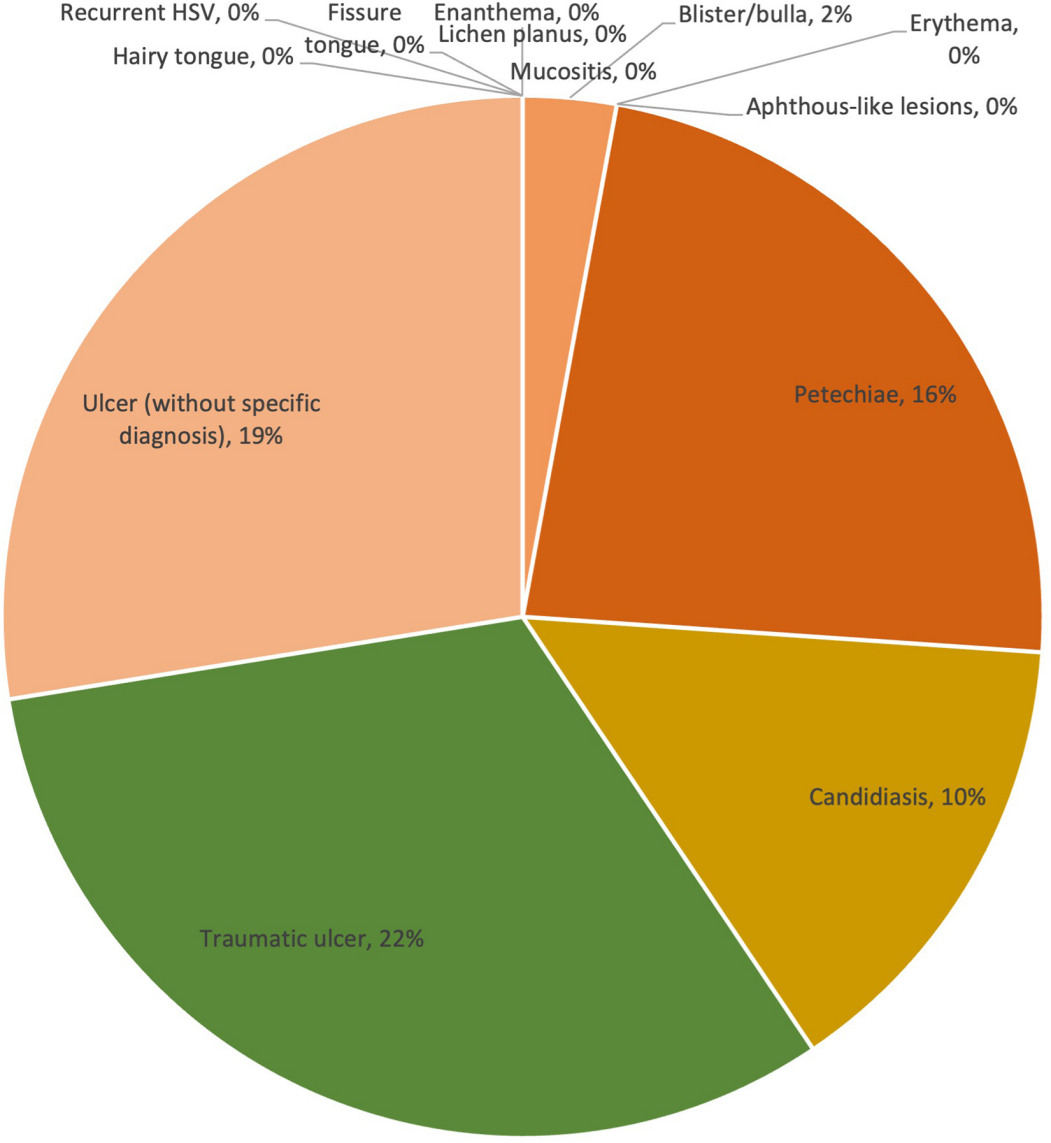
Prevalence of lesions in severe COVID-19 cases.

Within the severe COVID-19 group, ulcers without specific diagnosis remained to be one of the most common lesion types (19%). However, traumatic ulcers (22%), which were not observed in mild or moderate cases, became the most common lesion type in severe COVID-19 cases. Similar to traumatic ulcers, the prevalence of petechiae increased significantly from 2% to 3% (mild and moderate) to 16% (severe). Candidiasis (10%) and blister/bulla (2%) were also presented in severe cases, other types were not reported. The prevalence of lesion diagnosis/clinical presentation is shown in Figure 5.

#### 3.3.5. Statistical analysis of ulcers without specific diagnosis

**Figure 6:**
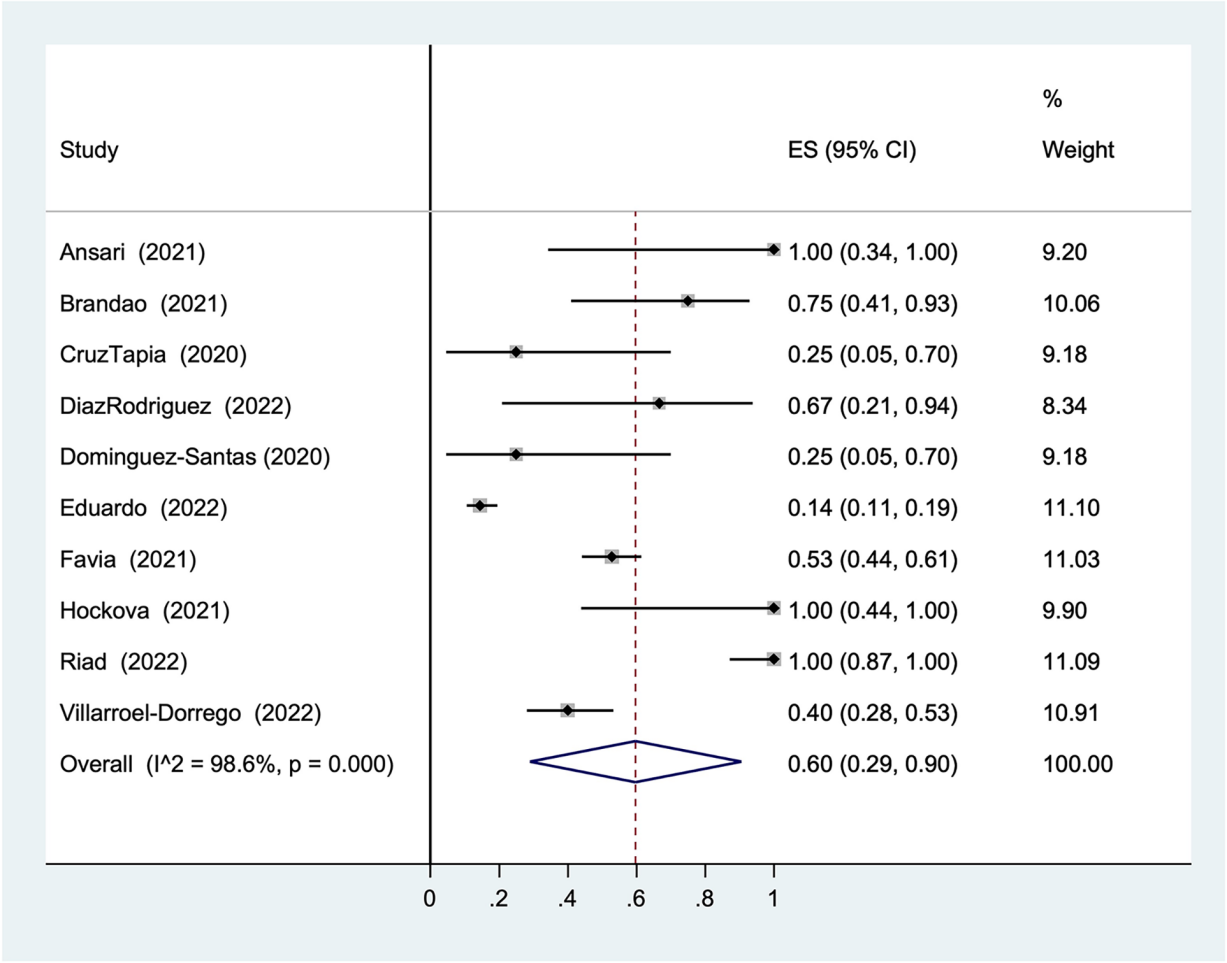
Prevalence of unpacifically diagnosed ulcer in COVID-19 patients.

Considering the distinguishing high proportion of ulcers without specific diagnosis in the result (30%), a statistical analysis was performed to investigate its overall prevalence in COVID-19 patients. The overall prevalence was 60% (95% CI: 29%–90%, *I*^2 ^=^ ^98.6%, Figure 6).

## 4. Discussion

### 4.1. Ulcers without specific diagnosis

Ulcers are defined as the breaks in the continuity of the epithelial covering of the body, followed by inflammatory changes in the exposed connective tissue (67). They are commonly caused by infectious or non-infectious conditions, and also act as an oral manifestation of systemic diseases or an indication of internal malignancy (68).

Although the overall result of the statistical analysis is still statistically significant (60%), the result does not show homogeneity of the data. Given the range of prevalence from 28% to 96% in 95% CI with 98.6 *I*^2^, the randomness of the results is indicated. Therefore, the results cannot support the hypothesis of an association between COVID-19 and ulcers without specific diagnosis.

### 4.2. Traumatic ulcers, candidiasis, petechiae and aphthous-like lesions

In the collected results, traumatic ulcers (10%), petechiae (9%), candidiasis (9%) and aphthous-like lesions (7%) have a similar prevalence in patients with COVID-19. As the severity of COVID-19 increased, aphthous-like lesions decreased substantially from 25% to 0%, whereas traumatic ulcers, petechiae and candidiasis all followed the increasing trend.

#### 4.2.1. Traumatic ulcers

Traumatic ulcers are superficial ulcerations that are associated with acute or chronic injury to the oral mucosa (67). The source of irritation is often found adjacent to the ulceration, indicating the cause of the lesion. Once the source of irritation is removed, the ulcer usually resolves within days.

According to the synthesized result, traumatic ulcers were only recognized in severe COVID-19 cases (60/277, 22%) when patients were in intensive care (ICU). It is common for patients in ICU to face life-threatening situations, such as severe pneumonia or even respiratory failure. As such, they are likely to be intubated for oxygenation, thereby resulting in traumatic ulcers at the intubation sites. Therefore, intubation in the course of treatment may be associated with the occurrence of traumatic ulcers, thus explaining their high prevalence in severe COVID-19 cases. Interestingly, in line with our findings, a recent study (69) also demonstrated a possible association between the intubation process in COVID-19 patients and traumatic ulcers. The cohort study analyzed COVID-19 patients with oral mucosal changes associated with hospitalization and found a statistical association between intubation and oral lesions. Taken together, a positive association between the incubation process and traumatic ulcers in COVID-19 patients can be considered.

#### 4.2.2. Aphthous-like lesions

Aphthous lesions are a common type of recurrent ulcer of infectious or non-infectious nature (68). It is believed that the mucosal destruction is the result of the production of tumor necrosis factor-alpha (TNF-α), which is caused by a T cell–mediated immunologic reaction (67). Its causative agents vary in different subgroups of patients, making it difficult to trace its etiology.

According to the synthesized result, aphthous-like lesions are mainly reported in mild COVID-19 cases (32/126, 25%) and less frequently in moderate and severe cases, with only 8 and 1 cases reported respectively. However, as aphthous-like lesions can be caused by a wide range of causative agents and it is difficult to associate the exact causative agent with COVID-19, it is difficult to substantiate the possible association between COVID-19 and aphthous-like lesions.

#### 4.2.3. Petechiae

Petechiae are classified as small hemorrhages in the skin, mucosa or serosa (67). It can result from repeated or prolonged elevation of intrathoracic pressure associated with many activities, such as repeated coughing or vomiting, or from non-traumatic causes, such as anticoagulant therapy or thrombocytopenia.

According to the synthesized result, petechiae are observed in mild (2%), moderate (3%) and severe (16%) COVID-19 cases, with a significantly higher prevalence in the severe group. Interestingly, a recent review (12) suggested that petechiae in COVID-19 patients with thromboembolic phenomena could be a consequence of anticoagulant therapy. It should be noted that thromboembolic phenomena are common complications in intensive care, so our finding of higher prevalence in severe cases (ICU patients) is consistent with the suggested association. However, this view remained a hypothesis because the reports included in the review did not indicate whether the patients were receiving anticoagulant therapy. Therefore, the hypothesis is not supported by evidence and more research is needed before a conclusion can be drawn.

Furthermore, it should not be neglected that petechiae only describe the clinical feature of the lesions observed, with an enormous variety of possible causes. Without differential diagnosis, the causative agents cannot be determined and therefore the correlation between petechiae and COVID-19 is still unclear.

#### 4.2.4. Candidiasis

Candidiasis is classified as a fungal infection caused by Candida species (67). Innocuous Candida carriage is thought to be associated with the yeast form, and only the invasive hyphal form causes disease. As Candida is part of the commensal oral microflora, candidiasis is recognized as a “disease of the diseased”. This means that the disease is the result of an interaction between the host and the organism, and that the switch of the organism into a pathogenic form is rather related to the immune status and the oral mucosal environment of the host.

According to the synthesized result, candidiasis is only detected in patients with moderate (14%) and severe (10%) COVID-19 cases. As the patient was hospitalized (moderate) or in intensive care (severe) and showed more advanced symptoms of COVID-19 infection, it is more likely that the patient's immune system is altered. In support of this hypothesis, a recent study (27) found that COVID-19 patients had reduced upregulation of CD80 on monocytes and abrogated release of IL-6, TNF, IL-1a and IL-1b in response to Candida albicans. Therefore, the observation of the prevalence of candidiasis is consistent with their histopathological findings, suggesting that the occurrence of candidiasis in COVID-19 patients may be due to the impaired host immune system.

### 4.3. The others

Similar to petechiae, erythema (5%), blister (4%), mucositis (2%) and enanthema (1%) only describe the clinical feature of the oral mucosal lesion, which may have numerous causes in different clinical situations. Thus, without a differential diagnosis, the possible association between them and COVID-19 infection is difficult to investigate.

Lichen planus is a common T cell-mediated immune reaction to an unknown trigger that results in cutanemucosal lesions (67). With a prevalence of 2% in the synthesized result, which is similar to the prevalence of oral lichen planus in the population (70), it is difficult to relate lichen planus to COVID-19.

Fissure tongue, which is thought to be strongly associated with heredity, is a condition characterized by the presence of grooves or fissures on the dorsal surface of the tongue (67). Due to its congenital nature, it is unlikely that COVID-19 is associated with its occurrence.

Hairy tongue is defined as a hair-like appearance on the dorsal tongue caused by the accumulation of keratin on the filiform papillae. It may be associated with smoking, general debilitation, poor oral hygiene, xerostomia-inducing medications, and a history of head and neck radiation therapy (67). As it is more associated with the medical history and behavior of the host, similar to fissure tongue, it is difficult to make an association with COVID-19.

### 4.4. Limitation of evidence

As shown in Table 1, most of the included studies were case reports (15/30) or case series (10/30), which lack control and exposure groups for scientific analysis, and most of them have a moderate (20/30) to high (5/30) risk of bias. Consequently, the source of these studies may be inaccurate or incomplete, resulting in heterogeneity of data. Hence, it is of low possibility to conclude the association between COVID-19 and oral mucosal lesions based on the available studies.

The previous review either only included systematic reviews which were not the primary article (71) or only summarised the present of oral lesions among all oral manifestations of COVID-19 (3, 7, 11). Therefore, the present review provides a detailed update on the prevalence of oral mucosal lesions presented in COVID-19 cases, especially an analysis of the oral mucosal lesion presentation pattern in different COVID-19 severity cases, identifying the possible causes of some oral mucosal lesions presented in COVID-19 patients.

The included case reports and case series, despite having a low level of evidence, clearly identified and reported the oral mucosal features presented in COVID-19 cases. Although lacking higher level studies, such as clinical trials, the summarised result can still provide a clinically relevant presentation on the types of lesions that are presented in COVID-19 patients and their possible associations, therefore providing a reference for clinicians.

Previous reviews have also demonstrated the common oral features of oral mucosal lesions in COVID-19-positive patients, and their corresponding hypothesis on the association between COVID and oral mucosal lesions. Some proposed that oral mucosal lesions may act as a secondary manifestation of COVID-19 (6, 10, 12, 17), or that COVID-19 disrupts the local microbiome or impairs oral mucosal immunity, causing oral mucosal lesions (12, 27, 72). Others pointed out that it may be caused by poor oral hygiene practice or intubation process during hospitalization, or adverse effects of COVID-19 medication (15, 69). However, similar to the findings of this systemic review, most of them (3, 5, 16, 20, 21, 23, 24) still cannot conclude that COVID-19 has an association with oral mucosal lesions due to the lack of detailed reports.

Therefore, combining the information from previous reviews and our results, further well-designed case-control studies are needed to analyze the association between COVID-19 and oral mucosal lesions and its possible mechanism.

## 5. Conclusion

This systematic review has updated the current knowledge on COVID-19 and oral mucosal lesions by identifying and summarising the common oral mucosal lesions and their prevalence in COVID-19 patients. Ulcers without specific diagnosis and aphthous lesions are unlikely to be associated with COVID-19, whereas traumatic ulcers, petechiae and candidiasis may be associated with COVID-19 due to the process of COVID-19 treatment or immune system impairment caused by COVID-19 infection. However, given the limited evidence from currently available studies, the association between COVID-19 and oral mucosal lesions remains difficult to clarify. The need for further studies has been demonstrated and emphasized. Health professionals should raise awareness of oral mucosal lesions in COVID-19 patients and provide appropriate treatment when necessary.

## Data Availability

The original contributions presented in the study are included in the article/Supplementary Material, further inquiries can be directed to the corresponding author.
